# Zeaxanthin: Metabolism, Properties, and Antioxidant Protection of Eyes, Heart, Liver, and Skin

**DOI:** 10.3390/antiox8090390

**Published:** 2019-09-11

**Authors:** Ana Gabriela Murillo, Siqi Hu, Maria Luz Fernandez

**Affiliations:** 1 Biochemistry Department, University of Costa Rica, San Pedro de Montes de Oca 2060, Costa Rica; anagabriela.murillo@ucr.ac.cr; 2 Department of Nutritional Sciences, University of Connecticut, Storrs, CT 06269, USA; siqi.hu@uconn.edu

**Keywords:** zeaxanthin, bioavailability, transport, antioxidant, age-related macular degeneration, liver health, atherosclerosis, inflammation

## Abstract

Zeaxanthin, a non-provitamin A carotenoid that belongs to the xanthophyll family, has been less studied than its isomer lutein. However, zeaxanthin has also been shown to have a number of beneficial effects for human health due to its ability to quench free radicals, exert antioxidant effects, as well as decrease inflammation. It is the purpose of this review to discuss the metabolism of zeaxanthin, including digestion, absorption, transport, and uptake by tissues, as well as the dietary or other factors which affect zeaxanthin bioavailability. In addition, this review also focuses on specific effects of this carotenoid on eye, skin, liver, and cardiovascular health. Data derived from human interventions, animal models of research, and in vitro and cell studies are discussed in this review.

## 1. Introduction

Zeaxanthin (β,β-Carotene-3,3′-diol), with a molecular weight of 568.8 Daltons and 11 conjugated double bonds, is a carotenoid that belongs to the xanthophyll family. These conjugated bonds are distributed between the polyene chain and the ionone rings. The ionone rings contain a hydroxyl group that can attach to fatty acids during esterification [1]. Lutein is the stereoisomer of zeaxanthin, and both of these carotenoids are generally found together in plant sources including green leafy vegetables, where the content can be as high as 40 mg per 100 g, while in the yellow-orange fruit and vegetables such as carrots, papaya, orange, and peaches, the concentration is less than 1 mg per 100 g. These xanthophylls can also be found in animal products, where they are more bioavailable because they are incorporated into the lipid matrix of the food, as is the case of egg yolks and cheese. Wheat can also be considered a source of zeaxanthin and lutein, mainly because wheat products are a common staple in some parts of the world [2]. Neither of these xanthophylls possess provitamin A activity.

Zeaxanthin has three stereoisomeric forms (i.e., (3R,3′R)-zeaxanthin, (3R,3′S)-zeaxanthin, and (3S,3′S)-zeaxanthin). The main form of zeaxanthin in nature is (3R,3′R)-zeaxanthin, while (3R,3′S)-Zeaxanthin, also called meso-zeaxanthin, is absent from dietary sources, the liver, or in circulation, but it can be found in ocular tissues [3]. It is important to mention that humans are unable to synthesize zeaxanthin and therefore it needs to be obtained from dietary sources [4].

It is recognized that both lutein and zeaxanthin predominantly accumulate in the retina, and the concentrations increase gradually towards the center of the macula, where it can be approximately 1000-fold higher than in other tissues [5]. Their presence in eyes is relevant as they have been shown to protect against age-related macular degeneration (AMD) [6] and cataracts. In addition, zeaxanthin has been shown to protect against oxidative stress in different tissues as well as systemic inflammation.

## 2. Factors Affecting Zeaxanthin Bioavailability

Carotenoids are normally very poorly absorbed. Absorption varies widely depending on other dietary components present in the meal and can fluctuate from 5–50%. Several factors have been identified as having important effects on zeaxanthin availability, including thermal processing, esterification, and some dietary components such as lipids, polysaccharides, and minerals.

### 2.1. Thermal Processing

Thermal processing can have both beneficial and deleterious effects on zeaxanthin bioavailability during food preparation. For example, it can damage the content of zeaxanthin in raw food, while it can also facilitate the release and solubilization of zeaxanthin for greater bioavailability [7]. During food processing, a high temperature may substantially reduce the concentrations of zeaxanthin, as has been reported in sweet corn, where canning at 121 °C decreased zeaxanthin content by 29% [7]. The loss of zeaxanthin can also vary due to the temperatures used for cooking [8]. For example, in comparison with raw egg yolk, zeaxanthin decreased by 11.6%, 5.9%, and 6% in boiled, microwaved, and fried eggs, respectively [8].

Thermal treatments, however, can also increase the release and bioavailability of zeaxanthin. For example, pasteurization at 98 °C for 21 s of an orange-carrot juice mixture increased the concentration of zeaxanthin by 45%, which may be associated with a disruption in the food matrix, promoting a better extraction of the carotenoid [9]. It has also been shown that wolfberries homogenized into hot skimmed milk are much more bioavailable compared with those in warm skimmed milk, with a three-fold increase of zeaxanthin with the hot milk [10], indicating that the higher temperature facilitates the incorporation of zeaxanthin into mixed micelles, thus increasing zeaxanthin bioavailability.

Bioavailability of carotenoids appears to be higher in the trans-isomers compared with the cis-counterparts [11]. Although some studies suggest that conversion of cis to trans form occurs in zeaxanthin, the impact of this conversion on bioavailability is still not clear [12].

### 2.2. Esterification

Most zeaxanthin in foods exists esterified as zeaxanthin di-palmitate [11], which needs to be hydrolyzed before incorporation into chylomicrons [13]. Yet, esterified zeaxanthin has been shown to have higher bioavailability than the free form. To evaluate which form was more bioavailable, 12 healthy subjects were administered 5 mg of free or zeaxanthin di-palmitate dissolved in palm oil and blended into yogurt, in a crossover design [14]. Supplementation with the esterified zeaxanthin resulted in two-fold greater mean area under the curve values compared with supplementation with the free form [14]. A more recent study using 16 healthy volunteers [15] also supported this finding. Participants consumed an aggregated zeaxanthin or zeaxanthin di-palmitate (both 6 × 10^−5^ M) in starch-based particles suspended in apple juice/apple puree, which was ingested with a balanced breakfast. The bioavailability of di-palmitate zeaxanthin was 23% higher than that of free zeaxanthin, as assessed in the circulating triglyceride-rich lipoproteins However, the underlying mechanism by which esterified zeaxanthin has enhanced bioavailability due to esterification needs to be clarified.

### 2.3. Dietary Polysaccharides

The bioavailability of zeaxanthin is affected by the type of polysaccharides in meals. For instance, a recent report indicated that zeaxanthin exhibits different bioavailability whether consumed in a starch-based or an alginate-based matrix [16]. In this study, healthy volunteers consumed a single dose of 20 mg of zeaxanthin with either a starch matrix (SMZ) or a cross-linked alginate matrix (AMZ) orally. Twenty-four hours after the administration of AMZ, the mean values of plasma total zeaxanthin and all-trans-zeaxanthin concentrations were lower than the basal levels; however, the values remained higher than basal levels with the SMZ group [16]. These results suggested that zeaxanthin in a starch-based matrix has a higher bioavailability than the one in an alginate-based matrix. Alginate decreases the permeability of intestinal mucus and inhibits lipid absorption, which may be responsible for the reduced bioavailability [17].

### 2.4. Dietary Lipids

As a carotenoid, zeaxanthin is also a lipophilic compound. Its absorption partially depends on bile acid solubilization and micelle incorporation. Dietary fats are considered to be essential for stimulating bile production in the intestine and subsequent micelle formation [18]. Although no study has specifically looked into the impact of fat amount on zeaxanthin bioavailability, it has been reported that dietary fat increased lutein bioavailability in human subjects [19]. In comparison to subjects receiving lutein supplementation in a low-fat (fat content ~3%, *w*/*w*) spread, the high-fat spread (fat content ~80%, *w*/*w*) group had a higher increase in serum lutein levels (158 ± 25 nM vs. 365 ± 38 nM) [19].

Moreover, Handelman et al. suggested that the type of dietary lipids also affects zeaxanthin bioavailability [20]. In this cross-over study, eleven moderately hypercholesterolemic men and women were recruited to consume egg yolk along with a beef tallow or a corn oil diet. The beef tallow group had a higher increase in plasma zeaxanthin compared with the corn oil group when compared with the baseline values (142% vs. 114%, respectively), indicating that compared with polyunsaturated fatty acids (PUFA), saturated fatty acids (SFA) led to higher zeaxanthin bioavailability. Gleize et al. also confirmed this finding [21] in a study where they evaluated lutein and zeaxanthin bioavailability by three methods: in vitro, with Caco-2 cells, and with orally administered rats. In the case of rats, when they consumed either a spinach-butter or a spinach-palmitic acid emulsion, they had greater concentrations of these carotenoids than when they consumed spinach with olive oils or fish oils. SFA-rich oil treatment also resulted in smaller mixed micelle formation, which was inversely related to the greater bioaccessibility measured in vitro [21].

### 2.5. Food Matrix

The food matrix is also an important factor for carotenoid bioavailability. Foods where zeaxanthin is in the lipid matrix of the food will be more easily absorbed, as is the case of eggs. When compared with only egg whites, whole eggs have substantially increased plasma zeaxanthin in children [22], young adults, [23,24], elderly people [25], overweight/obese individuals [26], and participants classified with metabolic syndrome [27]. Studies that have been conducted to elucidate the factors affecting the bioavailability of zeaxanthin are summarized in Table 1.

## 3. Absorption and Metabolism of Zeaxanthin

Zeaxanthin is a lipophilic compound and therefore is insoluble in aqueous media. However, zeaxanthin possesses two hydroxyl groups with a higher polarity compared with other carotenoids, suggesting that zeaxanthin might be absorbed and transported differently. It is important to understand zeaxanthin release, absorption, transportation, and distribution into tissues to have a better understanding of its biological functions.

Zeaxanthin needs first to be released from the food matrix, a process that starts in the stomach by the action of acid and digestive enzymes. Other carotenoids have been shown to be partially released from the food matrix in the stomach [28]. Similarly, zeaxanthin will solubilize into lipid droplets that convert to lipid emulsion particles smaller in size [1]. The distribution of carotenoids in emulsion particles is based on their polarity, thus those with lower polarity would migrate to the lipid core of the particles, while those with higher polarity (e.g., zeaxanthin) will locate in the surface where proteins and phospholipids are found [29].

### 3.1. Digestion in the Intestinal Lumen

Lipid emulsion particles containing zeaxanthin and other carotenoids move from the stomach to the duodenum. Dietary fat present in the emulsion stimulates the gall bladder to secrete bile acids and triggers the release of lipases from the pancreas. All enzymes produced by the pancreas hydrolyze the food matrix and promote the release of zeaxanthin. Pancreatic lipases also help in the transfer of zeaxanthin from the emulsion particles to the lipid phase of the micelles [14].

Zeaxanthin is present in plants in the esterified form [30]; however, this xanthophyll is found in the free form in chylomicrons and other lipoproteins [31], indicating that the esterified zeaxanthin needs to be hydrolyzed before it is transported into the lymph or blood. Carboxyl ester lipase, one of the dietary fat-induced pancreatic lipases, has been found to hydrolyze esterified carotenoids [32]. A study conducted in vitro in which mixed micelles containing zeaxanthin were incubated with carboxyl ester lipase reported that after incubation there was an 84% decrease in esterified zeaxanthin, with a proportional increase in the free form [32]. These results suggested that carboxyl ester lipase may also be responsible for the de-esterification of zeaxanthin in the intestine.

### 3.2. Absorption by Enterocytes

The concentration gradient of zeaxanthin between the enterocyte and the cell membrane will control the rate of passive diffusion, which is the most accepted approach by which carotenoids are absorbed into the small intestine [33,34]. The mixed micelles directly contact and diffuse into the membrane of enterocytes, releasing carotenoids including zeaxanthin into the cytoplasm of enterocytes [33].

However, there is more recent evidence showing that xanthophylls can also be transported into enterocytes by receptors including scavenger receptor class B type 1 (SR-BI) and Niemann-Pick C1-Like 1 (NPC1L1). For example, it has been shown that treatment of Caco-2 cells with SR-B1 inhibitor decreased cellular uptake of lutein by 57% [35]. Also, the inhibition of NPC1L1 by ezetimibe at 40 µM decreased lutein uptake by 40% [36].

### 3.3. Efflux from Enterocytes

There are two different pathways by which zeaxanthin may efflux from the enterocytes, one of which is the assembly into chylomicrons and release into the lymph [37], or it can be secreted into the lymph or the portal vein from the enterocytes within the small intestine-derived high density lipoprotein (HDL) [38]. For the former pathway, zeaxanthin along with other carotenoids and lipid molecules is assembled into nascent chylomicrons in the Golgi apparatus via the action of microsomal transfer protein (MTP), and after the incorporation of B-48, it is released into the lymphatics [39]. The mechanism by which xanthophyll intracellular translocation occurs in the enterocyte has not yet been reported. Because zeaxanthin is a lipophilic molecule, it could possibly be transported by intracellular binding protein in enterocytes of which retinal lutein-binding protein and steroidogenic acute regulatory domain 3 could be good candidates [39,40], although their expression in enterocytes is not known.

Research on carotenoid lipoprotein distribution has shown that lutein and zeaxanthin are predominantly found in HDL (53%), while these proportions are lower in low density lipoprotein (LDL) and very low density lipoprotein (VLDL) [27], therefore the hypothesis that they can be directly incorporated into HDL from the enterocyte is valid. Moreover, the transporter ATP-binding cassette AI (ABCA1) can be involved in this efflux process. Niesor et al. reported that induction and inhibition of ABCA1 expression, via liver X receptor (LXR) agonist T0901317 or statin treatment respectively, resulted in increased or decreased absorption of dietary lutein and zeaxanthin in a hamster model [33]. Furthermore, inducing ABCA1 expression using LXR agonist increased lutein secretion only in the presence of apolipoprotein A1 (ApoA1) in Caco-2 cells [39]. These results suggest that ABCA1 is responsible for transferring lutein and zeaxanthin from the basolateral surface of enterocytes toward ApoA1 in nascent HDL.

### 3.4. Transport in Blood

Following efflux, chylomicrons travel through the lymphatic system and reach systemic circulation at the thoracic duct, where they transport zeaxanthin along with other carotenoids and dietary lipids to the extrahepatic tissues. Chylomicrons in circulation are rapidly hydrolyzed by lipoprotein lipase bound to the capillary endothelium, converting them into chylomicron remnants that are later removed by the liver. Because carotenoids follow the same pathways as lipids, it is possible that part of zeaxanthin is taken up by extrahepatic tissues. After uptake by the liver, zeaxanthin can be stored, eliminated in the bile, or re-distributed to extrahepatic tissues via VLDL secretion [41]. Zeaxanthin and other carotenoids in VLDL are distributed into intermediate density lipoprotein (IDL) and LDL following the delipidation cascade. Zeaxanthin in LDL is taken up by the LDL receptor or LDL receptor-related protein (LRP).

Both LDL and HDL are primary transporting vehicles of xanthophylls in the bloodstream. However, the exact source of HDL zeaxanthin is not fully elucidated. Not only the intestine, but also extrahepatic tissues and triglyceride-rich (TG) lipoproteins may contribute to the HDL zeaxanthin pool. Tyssandier et al., for example, have demonstrated that lutein can be exchanged between TG-rich lipoprotein and HDL via cholesterol ester transfer protein and lecithin cholesterol acyltransferase [42]. This evidence indicates that zeaxanthin in HDL may partially derive from TG-rich lipoproteins including chylomicron and VLDL.

### 3.5. Tissue Distribution

The major sites of carotenoid storage are the liver and the adipose tissue, but they can also be found in kidney, adrenals, and testes [43]. Few studies, if any, have specifically looked into the distribution of zeaxanthin among different tissues. Since lutein and zeaxanthin are absorbed and transported similarly, accumulation of lutein and zeaxanthin in different tissues might follow a similar pattern. Recently, the distribution of lutein in tissues was studied [44]. The highest accumulation was observed in eyes, followed by adrenal gland and liver. In adipose tissue, lutein levels were found to be lower compared with plasma. Finally, lutein was also found in the kidney, heart, and brain [45].

### 3.6. Uptake of Zeaxanthin by the Eyes

In the peripheral part of the retina, there is a 2:1 lutein:zeaxanthin ratio, while zeaxanthin becomes the dominant pigment in the macular area [5]. Furthermore, at the center of the macula, total zeaxanthin is composed of (3R,3′R)-zeaxanthin, the principal form of zeaxanthin in nature, and meso-zeaxanthin at a 1:1 ratio [5]. This change in the predominant macular carotenoids could be explained by the conversion of meso-zeaxanthin from lutein, as was later demonstrated with an experiment using a cell model [45].

Both HDL and SR-BI, a cell surface glycoprotein that functions as an HDL receptor, may be involved in xanthophyll uptake by the eyes. Connor et al. examined the role of HDL in xanthophyll delivery to the retina by using Wisconsin hypoalpha mutant chicken, an animal model of HDL deficiency [46]. After being fed a diet high in lutein, chickens increased the levels of this carotenoid in plasma, heart, and liver instead of in retina, indicating that HDL is essential for delivery of xanthophylls to the retina [47].

Evidence shows that lutein and zeaxanthin are preferentially taken up by human retinal pigment epithelial (RPE) cells via an SR-BI-dependent mechanism [47]. In addition, when ARPE-2 cells, a human RPE cell line, were treated with lutein, zeaxanthin, or β-carotene, the quantities of lutein and zeaxanthin absorbed by the cells were two-fold higher than that of β-carotene [48]. SR-BI knockdown via small interfering RNA (siRNA) or blocking by an antibody significantly decreased the cellular uptake of the xanthophylls, especially zeaxanthin [48].

**Figure 1:**
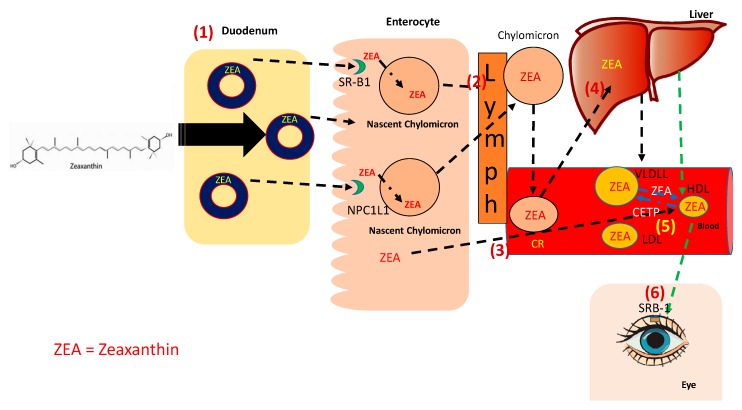
Metabolism of Zeaxanthin. Absorption, Uptake, and Trafficking: (**1**) Esterified zeaxanthin (ZEA), after being hydrolyzed, is incorporated into the micelles [14] and can be taken up by the enterocyte by two receptors, SR-B1 and NPC1L1 [37]. ZEA can have two fates in the enterocyte: (**2**) it can be incorporated into the chylomicron via the action of microsomal transfer protein and be released to the lymphatics [38] or (**3**) can be directly incorporated into nascent HDL. (**4**) As the chylomicron is being delipidated by lipoprotein lipase, some ZEA can be taken up by the peripheral tissues and the rest return to the liver as chylomicron remnant [37]. (**5**) ZEA can also be part of HDL by the interchange of lipids between HDL and the TG-rich lipoproteins [43]. (**6**) ZEA is taken by the eye by SR-B1 via HDL [47]. SR-B1: scavenger receptor class B type 1. NPC1L1: Niemann-Pick C1-Like 1.

Digestion, absorption, transport, uptake, and tissue distribution of zeaxanthin are depicted in Figure 1.

## 4. Effects of Zeaxanthin in Human Health

### 4.1. Antioxidant Properties of Zeaxanthin

Carotenoids play essential roles in protecting cellular membranes and lipoproteins against reactive oxygen species (ROS)-induced oxidative stress [48]. Results from in vitro and in vivo studies involving animal models and human have demonstrated that lutein and zeaxanthin potentially protect against chronic eye and cardiovascular diseases, such as age-related macular degeneration (AMD), cataract, coronary heart disease, and stroke [49]. Further, the hydrophilic properties of zeaxanthin may enhance its antioxidant properties in aqueous media, such as in the blood circulation. In human plasma, loss of zeaxanthin upon photo-irradiation in the presence of photo-oxidation sensitizer methylene blue was faster than that of β-carotene and lycopene [50]. The data from this in vitro study indicate that zeaxanthin reacts with singlet oxygen more efficiently than nonpolar carotenoids in the water phase, probably due to its higher polarity [50]. Oxidative stress is involved in the initiation and progression of these chronic diseases [51,52]. Therefore, it is necessary to study the antioxidant effects of these two xanthophylls and the underlying mechanisms.

Zeaxanthin also protects rats against oxidative stress. For instance, lutein and zeaxanthin are able to protect rats against high-fat diet (HFD)-induced oxidative stress. HFD feeding for eight weeks significantly increased malonadelhyde (MDA) level while reduced total antioxidant capacity in the rat retina [53]. Gavage of lutein- and zeaxanthin-containing marigold flower extract (composed of 80% carotenoids, with 67% of lutein and 13.5% of zeaxanthin) at a dose of 100 mg/kg reduced HFD-induced MDA production and recovered total antioxidant capacity in the rat retina [53].

Zeaxanthin can exert its antioxidant property by directly quenching ROS. Similar to other carotenoids, zeaxanthin contains a chain of isoprene residues bearing conjugated double bonds. These double bonds allow carotenoids to receive the extra electron and uptake energy from excited molecules, followed by dissipation of the absorbed energy as heat in plants [54]. Moreover, zeaxanthin can also exhibit its antioxidant property by facilitating glutathione (GSH) synthesis in human RPE cells. Zeaxanthin treatment increased Nuclear receptor factor 2 (Nrf2) translocation by decreasing binding activity of Nrf2 to Kelch-like ECH-associated protein 1 (Keap1) in ARPE-19 cells [55]. Nrf2 then induced α-glutamyl-cysteine ligase expression, the rate-limiting enzyme regulating the synthesis of GSH, and thus increased the GSH amount in the cell. Furthermore, GSH synthesis inhibition by buthionine sulphoximine abolished the protective effect of zeaxanthin against oxidative stress-induced mitochondrial membrane potential reduction and cell apoptosis in ARPE-19 cells [55]. This evidence suggests GSH is critical for zeaxanthin to exert an antioxidant property in RPE cells.

According to Bhosale et al., binding with glutathione S-transferase (GSTP1), a zeaxanthin-binding protein in human eyes, increases antioxidant activity of zeaxanthin [56]. In the presence of GSTP1, both (3R,3′R)-zeaxanthin and meso-zeaxanthin exhibited better protection against peroxyl radical-induced lipid peroxidation in vitro compared with the groups without GSTP1 [56]. GSTP1 stabilizes zeaxanthin against degradation induced by peroxyl radicals, thereby enhancing its antioxidant property [56].

In comparison with lutein, zeaxanthin has only one additional conjugated double bond [57]. However, this small difference in the molecular structure still leads to a difference in their antioxidant activities. Evidence shows zeaxanthin is a more potent antioxidant than lutein. Compared with lutein, zeaxanthin showed better protection against photo-irradiation and singlet oxygen-induced in vitro degradation of A2-PE, a fluorophore in the photoreceptor outer segment membrane [58]. Zeaxanthin also showed a higher singlet oxygen quenching rate than lutein in vitro (2.3 × 10^8^ vs. 1.1 × 10^8^ M^−1^s^−1^, respectively) [59]. A possible explanation is that lutein exhibits greater aggregation in membranes than zeaxanthin, which leads to loss of singlet oxygen quenching [59]. It has been reported that the number of conjugated double bonds of carotenoids increases with their quenching efficiency of singlet oxygen, which may also be attributable to the differences between luein and zeaxanthin [57,59].

### 4.2. Lutein and Eye Health

Lutein and zeaxanthin are found along with long-chain PUFA in human rod outer segment membranes, in which oxidative stress is the highest [60]. The identification of β,β-caroten-3,3′-dione, 3′-Hydroxy-βββ-caroten-3-one, and 3-Hydroxy-β\β-caroten-3′-one as oxidation products of lutein and zeaxanthin in human retina also supports that these xanthophylls function as antioxidants in human eyes [61].

Age-related macular degeneration is the major cause of visual impairment and irreversible blindness among the senior population in the U.S. [62]. In 2010, the estimated number of AMD patients was 2.07 million, which is anticipated to increase by more than two-fold in the following forty years [62]. AMD may lead to vision blurry or loss of vision in patients’ central visual field, which can cause difficulty in reading, writing, driving, or other daily activities [62]. About two decades ago, antivascular endothelial growth factor (VEGF) treatment was introduced as an effective therapy, which can recover the vision of AMD patients [63]. Nevertheless, this treatment requires frequent intravitreal injections along with a high cost. Therefore, an alternative treatment that does not come with a steep price and is less invasive would be a better choice.

Several studies have investigated the relationship between AMD and retinal lutein and zeaxanthin levels in human subjects. For example, Bone et al. measured lutein and zeaxanthin concentrations in donated retinas from healthy controls and AMD patients (*n* = 56 for each group) [64]. They reported that compared with healthy subjects, the combined concentrations of lutein and zeaxanthin in three concentric regions of the macula were decreased in AMD patients. The AMD risk of subjects in the highest quartile of retinal lutein and zeaxanthin levels was 82% lower than those in the lowest quartile (odds ratio (OD) = 0.18; 95% confidence interval (CI) = 0.05–0.64) [64]. The data suggest that retinal xanthophyll content is negatively correlated with the risk of AMD.

A recently published article evaluated relationships among macular pigment optical density (MPOD), which represents macular pigment levels within the retina, and age among Irish subjects [65]. The researchers found a moderate age-dependent decline in the MPOD among 79 senior subjects (age of 65, on average, *r* = −0.251, *p* = 0.045). This trend still existed after adding 462 more subjects, in which a younger population was included (age ranged from 18 to 67, (*r* = −0.179, *p* = 0.000) [65]. Moreover, they also reported that MPOD in subjects without AMD onset was significantly higher than in subjects with early-stage AMD (average MPOD of 0.25 ± 0.17 and 0.14 ± 0.13, respectively). It is widely accepted that age is one of the major risk factors for AMD onset [65]. This study indicates that aging may lead to AMD onset by depleting retinal xanthophylls.

In addition to retinal xanthophyll contents, plasma xanthophyll concentrations are also negatively associated with AMD risk in humans. The association between the risk of AMD and plasma xanthophyll levels was investigated among 380 elderly subjects in the U.K [66]. Subjects with plasma zeaxanthin in the lowest tertile had a two-fold higher risk of AMD when compared with those in the highest tertile (OD = 2.0 and 1.0, respectively, 95% CI = 1.0–4.1). Compared with subjects in the highest tertile, the risk of AMD was also increased in subjects with the lowest tertile of plasma lutein (OD = 1.7 and 1.0, respectively, 95% CI = 0.9–3.2) [66]. However, in the case of lutein, the association was not statistically significant. Therefore, plasma xanthophyll levels can be an indicator of AMD risk, with plasma zeaxanthin level probably serving as a better indicator than plasma lutein level.

Supplementation of lutein and zeaxanthin has shown improvement in visual performance of patients with early age-related macular degeneration. Forty-seven patients with early AMD were supplemented with a combination of lutein and zeaxanthin (20 mg and 0.86 mg per day, respectively) or meso-zeaxanthin, lutein, and zeaxanthin (17 mg, 3 mg, and 2 mg per day, respectively) for 36 months [67]. Both dosages were equivalent to 22 mg of total macular carotenoids. Both supplementations significantly increased MPOD in patients compared with the basal level, along with a significant improvement in letter contrast sensitivity [67].

Another study by Seddon et al. suggested AMD risk reduced with increased dietary intake of lutein and zeaxanthin. In this case-control study, 356 patients diagnosed with the advanced stage of AMD and 520 control subjects aged 55 to 80 years were recruited [68]. Compared with subjects in the lowest quintile of dietary carotenoid intake, the risk for AMD of those in the highest quintile decreased by 43% (OD = 1.0 and 0.57, respectively; 95% CI = 0.35–0.92) [68]. Moreover, lutein and zeaxanthin intake had the strongest negative correlation with the risk for AMD among all different dietary carotenoids. These associations demonstrate that increasing dietary intake of lutein and zeaxanthin may decrease the risk of advanced AMD.

The lesion in the retinal pigment epithelium initiates development of AMD due to RPE cells death and degeneration [69]. During the development of AMD, cellular oxidative stress plays a vital role in RPE cell death [70]. The previous section demonstrated that zeaxanthin exhibits a strong antioxidant property by quenching ROS and inducing GSH production in vitro and thereby inhibiting oxidative stress-induced RPE cell apoptosis. Moreover, zeaxanthin can protect RPE cells by inhibiting lipofuscin production. Excessive oxidation also induces the formation of lipofuscin in the RPE, which is an undegradable complex of intracellular protein aggregation [71]. The accumulation of lipofuscin can lead to cellular toxicity and functional impairment of RPE cells, which may be involved in AMD development [72].

Zeaxanthin treatment has also been shown to decrease the accumulation of lipofuscin formed in cultured RPE cells and in vivo. Treatment of lutein or zeaxanthin for two weeks significantly reduced lipofuscin accumulation induced by normobaric hyperoxia in rabbit or calf RPE cells compared with the control group [73]. Moreover, zeaxanthin treatment in rabbit RPE cells had a greater reduction than lutein treatment [73]. In addition, Bhosale reported the inhibition of N-retinylidene-N-retinylethanolamine (A2E), one of the essential components of lipofuscin, production in the RPE of Japanese quail by xanthophyll supplementation [74]. For xanthophyll supplementation, the quails were gavaged daily with a microbial extract rich in lutein or zeaxanthin for 16 weeks (equivalent to 0.2 mg of carotenoid per bird per day) [74]. A2E levels rose more than 6-fold relative to basal levels in the control group during the treatment period (due to aging), while this increase was diminished in the lutein- or zeaxanthin-supplemented quails [74]. All this evidence suggests zeaxanthin may protect against AMD by inhibiting lipofuscin production.

Light exposure, especially blue light (wavelength ranged between 400 and 500 nm), has been hypothesized as a risk factor for AMD. An age-matched control study among 838 watermen revealed patients with advanced AMD had significantly higher exposure to blue light over the past 20 years [75]. Similar to other carotenoids, the presence of a conjugated polyene chain in the zeaxanthin molecule allows it to absorb light [76]. The maximum light absorbance of zeaxanthin is between 445 and 472 nm in dichloromethane and 451 nm in ethanol, which falls in the blue light wavelength range [63,77]. Therefore, zeaxanthin can function as a blue light filter and reduce the intensity of blue light that reaches the retina and thus protect against AMD development [64]. Supplementation with pure zeaxanthin (2.2 mg/kg/d) for 22 weeks significantly reduced the area of lesion induced by acute blue light exposure in the fovea of monkeys, which supports this hypothesis [78].

### 4.3. Zeaxanthin and Liver Diseases

Nonalcoholic fatty liver disease (NAFLD) is a metabolic disorder which involves lipid accumulation in the hepatocytes and has been linked to obesity and diabetes, and can progress to more severe pathologies, being hepatocellular carcinoma the most aggravated condition [79]. NAFLD has been recognized as recurrent liver disease in Western countries, and the predictions are that it will be associated with liver transplantation in the near future [80,81].

It has been reported that oxidative stress plays a pivotal role in the development and progression of NAFLD, mainly because the liver is a major organ attacked by ROS. Liver cells are the most sensitive to oxidative stress [82]. Oxidative stress may lead to mitochondrial dysfunction, which causes decreased fatty acid oxidation, and hence, incrementes lipid accumulation and impaired liver function [83,84]. For this reason, the use of antioxidants like zeaxanthin has been studied as a potential mechanism of preventing and treating NAFLD [85].

Using data from the 2003–2014 National Health and Nutrition Examination Survey (NHANES), Christensen et al. found that prevalence for NAFLD among participants was 33%. Intakes of all carotenoids, except lycopene, were found to be lower in this group than in the group without NAFLD. This correlation was significant for the highest quartiles of intake (measured by a 24 h recall) of α-carotene, β-carotene, β-cryptoxanthin, and lutein/zeaxanthin [86].

The protective effects of carotenoids are not limited to NAFLD. Chamberlain et al. [87] used male Mongolian gerbils and found that zeaxanthin exerts a protective action against methionine–choline-deficient diets that induced NASH, which is considered an end-stage liver disease. In this study, the animals were divided into four groups: a control diet, and three dietary groups with methionine and choline deficiency containing 0, 12.5, or 25 mg/Kg of zeaxanthin, respectively. No major differences in histopathology were observed after 6 weeks between supplemented groups and controls. However, supplementation with 25 mg/kg of zeaxanthin reduced fibrosis and lipid peroxidation to baseline, which suggests that this carotenoid may be useful in the treatment of NASH.

Another pathology that has shown to be improved by carotenoids, specifically zeaxanthin, is alcoholic fatty liver disease (AFLD), which is the abnormal accumulation of triglycerides in the hepatocytes, with chronic alcohol intake as the primary cause. Just as with NAFLD, AFLD can progress to alcoholic steatohepatitis (ASH), hepatic fibrosis, cirrhosis hepatocellular carcinoma, and eventual death. Also, the pathogenesis of both diseases involves oxidative stress and inflammation, which suggests that antioxidant therapy could be useful [88,89]. To asses this, Xiao et al. [59] evaluated the therapeutic effects of zeaxanthin dipalmitate on a rat AFLD model.

In this study, 24 female Sprague-Dawley rats were randomly assigned to one of three groups, a control group, a fatty liver disease group challenged with alcohol, and a group to which 25 mg/Kg of zeaxanthin were added. The results of this study showed that the rats who were administered alcohol had a clear evidence of AFLD, while the treatment with zeaxanthin improved this condition and animals had lower oxidative stress and hepatic apoptosis. Authors suggested their results were associated with the modulation of the MAPK pathway [59].

Similar protective effects were reported by Gao et al. [90]. In this study, seven-week-old male Sprague-Dawley rats were fed a liquid diet, and one group had alcohol added progressively. After this period, rats were divided into four groups: control, ethanol, vehicle – zeaxanthin, and ethanol + zeaxanthin groups. The zeaxanthin (10 mg/kg) was gavaged every day from the start of the third week for another 2 weeks. As expected, the ethanol challenge caused an increase in serum alanine-amino transferase and aspartate amino transferase concentrations. The treatment with zeaxanthin effectively attenuated these histological changes by reducing accumulation of fat droplets and the infiltration of inflammatory cells. For these results, the authors concluded that zeaxanthin could be an effective ameliorative agent against ethanol-induced hepatic damage [90].

### 4.4. Zeaxanthin and Atherosclerosis

Atherosclerosis is an inflammatory disease that affects arterial walls. Its pathogenesis involves lipid and inflammatory cell accumulation within the intima, causing endothelial dysfunction and other metabolic alterations [91]. Atherosclerosis is the leading cause of cardiovascular disease (CVD), which is the number one cause of death in the western world [92,93].

It is widely accepted that lipoprotein metabolism plays a fundamental role in the progression of atherosclerosis. Particularly, several forms of LDL are considered atherogenic, such as small and dense LDL particles and oxidized LDL particles [94]. LDL oxidation caused by oxidative stress is considered crucial in the development of atherosclerosis. In fact, circulating oxidized LDL is considered a risk factor that can be used to identify individuals at risk for CVD [95,96,97]. Therefore, clinical strategies using food antioxidants such as carotenoids could be useful for the prevention or treatment of this disease [98].

As previously stated, lutein and zeaxanthin are mainly transported in HDL particles [27,99]. This specific transportation leads to bigger HDL particles, which are considered anti-atherogenic since they can carry more cholesterol from extra hepatic tissues, including foam cells, to the liver in the reverse cholesterol transport [100,101]. However, intervention studies also have shown that up to 22% of total xanthophylls are found in LDL particles [99]. This explains how these xanthophylls can also prevent atherosclerosis via inhibiting LDL oxidation.

Using a cell model, Carpenter et al. [102] tested the potential of three carotenoids, including zeaxanthin in preventing LDL oxidation, which was measured by electrophoresis, TBARS, and gas chromatography. The results showed that LDL oxidation was inhibited by each of the carotenoids in a concentration-dependent manner [102].

In humans, Kishimoto et al. [103] investigated the effects of adding one egg (an important source of lutein and zeaxanthin) per day to the diet of 19 male Japanese adults with mild hypercholesterolemia and found that consuming one soft boiled egg per day for 4 weeks in addition to their habitual diet increased their cholesterol intake without changing serum cholesterol or LDL concentrations, but these LDL particles were less oxidized. Also, the egg intake increased the participants’ serum lutein and zeaxanthin concentrations, which were inversely correlated to malondialdehyde-modified LDL, suggesting that dietary eggs could be considered atheroprotective because they increase the concentration of these oxycarotenoids in plasma [103].

In the Los Angeles Atherosclerosis study, a cohort of 269 women and 304 men were randomly selected from a group of employees and assessed for atherosclerosis risk factors at baseline and after 18 months. Researchers found an inverse association between plasma lutein and atherosclerosis in this sample [104]. Further on, they investigated the relationship of plasma antioxidants, including both lutein and zeaxanthin and carotid intima-media Thickness (IMT) using the same sample, and the results showed that an 18 month change in IMT was significantly inversely related to lutein and zeaxanthin [105].

Zou et al. [106] also assessed IMT and plasma carotenoids in The Beijing Atherosclerosis study. This was a case-control study with 125 subjects with early atherosclerosis and 107 controls aged 45–68 years. The researchers measured IMT and arterial stiffness using carotid ultrasonography and measured serum carotenoids with HPLC. In the cases, lutein serum concentration was significantly lower than that of the controls and inversely associated with IMT. On the other hand, zeaxanthin was negatively correlated with right common carotid artery stiffness, but there was no difference in zeaxanthin serum concentration between cases and controls, suggesting that the role of lutein in the prevention of atherosclerosis is clear, but zeaxanthin’s role may need further research [75].

### 4.5. Zeaxanthin and Skin Health

As mentioned before, lutein and zeaxanthin absorb blue light, which has the shortest wavelength of the visible spectrum (400–500 nm) and is highly oxidative and damaging since it can penetrate tissues such as the eye or the skin, producing ROS, inflammation, and mitochondrial dysfunction [107,108]. The protective properties of these lutein and zeaxanthin against blue light damage have been described widely regarding eye health, however similar filtering effects can be observed in the skin [109]. The skin’s natural antioxidant capacity is due to the presence of enzymatic antioxidants, vitamins, and carotenoids which overall protect the skin against overexposure of damaging lights, which in the eye or the skin can lead to ROS production, inflammation, and mitochondrial dysfunction [110].

One model to study the effects of damaging light is the hairless mouse skin. Using this model, Astner et al. [111] evaluated the effects of lutein and zeaxanthin against blue light damage. Twenty-four female mice were assigned to 0%, 0.04/0.03% or 0.4/0.03% lutein/zeaxanthin, respectively. After 2 weeks, the animals were irradiated with artificial ultraviolet light (UVB) light and assessed on the degree of ear swelling for inflammation, epidermal hyperproliferation, and inhibition of sunburn cell formation. The results of this study showed that 24 and 48 h after radiation, ear swelling was reduced in a dose-dependent manner by lutein and zeaxanthin supplementation. This also reduced epidermal hyperproliferation and the number of sunburn cells. These findings suggest that oral supplementation can protect against UVB light exposure [111].

**Figure 2:**
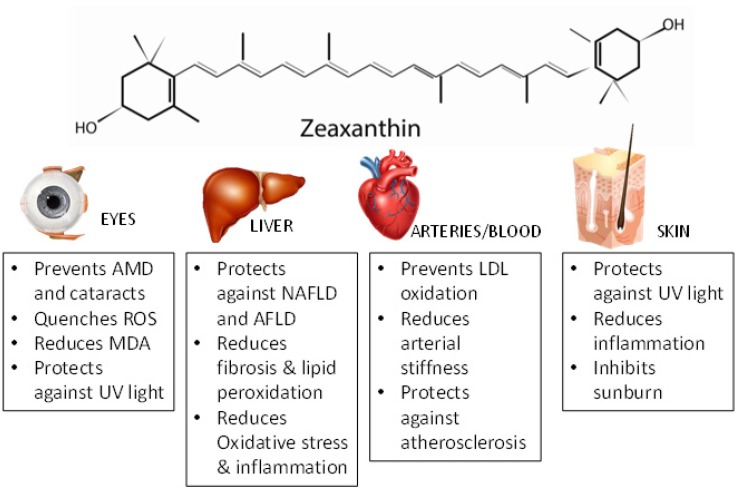
Protective Effects of Zeaxanthin on Eyes, Liver, Heart, and Skin. Zeaxanthin has been shown to have protective effects in eyes by preventing age-related macular degeneration (AMD) [64,65,66], quenching reactive oxygen species (ROS) [71], and protecting against UV light; In the liver, it protects against nonalcoholic fatty liver disease (NAFLD), alcoholic fatty liver disease (AFLD), reduces fibrosis and lipid peroxidation [87], and reduces oxidative stress and inflammation; in the arteries and blood, zeaxanthin prevents LDL oxidation [102], reduces arterial stiffness [104], and protects against atherosclerosis [106]; and in the skin, zeaxanthin protects against UV light [111], reduces inflammation [112], and inhibits sunburn [111]. MDA: Malonadlehyde.

In humans, Polombo et al. [112] conducted a study in females who had premature signs of skin aging. In this double-blind, placebo-controlled study, women consumed either a placebo or a supplement with 0.5 mg lutein and 0.3 mg zeaxanthin. Skin health parameters such as hydration, elasticity, lipid peroxidation, and photoprotective activity were evaluated several times during 12 weeks. The results showed that the supplementation group had a significant reduction of lipid peroxidation, improved skin hydration, elasticity, and photoprotective activity when compared with placebo-treated women. These results suggest that both lutein and zeaxanthin could exhibit protective effects against oxidative light in humans [112]. The main antioxidant effects in different organs/tissues are presented in Figure 2.

## 5. Conclusions

There is sufficient evidence documenting the importance of zeaxanthin as an antioxidant, especially in the eyes. There are also accumulated data demonstrating that zeaxanthin intake from supplement or dietary sources may protect against AMD development via its antioxidant and blue-filtering properties. However, it is still necessary to provide evidence to support that zeaxanthin reduces the risk of AMD onset or slows the progression of AMD in the long-term. Current zeaxanthin supplementation studies only showed the effect of zeaxanthin in years, instead of in decades [65,66]. Further, to have a better interpretation of the protective functions of zeaxanthin against AMD development, a complete understanding of the AMD pathophysiology is necessary. For instance, although this review highlights that zeaxanthin treatment inhibits lipofuscin accumulation, the potential role of lipofuscin in the development of AMD is not clearly elucidated.

Zeaxanthin has also been found in organs other than eyes, such as skin and brain [109,113], which strongly suggests that zeaxanthin supplementation may protect skin against UV light damage and may have a beneficial role on cognitive function in seniors [113]. However, the delivery of zeaxanthin to these two organs and subsequent intra-organ metabolism has not been reported. Future studies should address all these pending questions.

## Figures and Tables

**Table 1:** Factors affecting zeaxanthin bioavailability.

Factor	Bioavailability	Population Studied	Reference
**Thermal Processing**	↑ lower temperatures	In vitro	[7]
	↑ lower temperatures	In vitro	[8]
	↑ higher temperatures	In vitro	[9]
	↑ higher temperatures	In vitro	[10]
**Esterification**	↑ esterified vs. Free	12 healthy volunteers	[14]
**Esterification**	↑ esterified vs. Free	16 healthy volunteers	[15]
**Lipids**	↑ SFA vs. PUFA	11 hypercholesterolemic men	[20]
	↑ SFA compared to PUFA or MUFA	Rats	[21]
**Carbohydrates**	↑ SMZ vs. AMZ	48 healthy volunteers	[16]
**Eggs**	↑ eggs vs. egg substitute	224 volunteers including children, healthy young and old, overweight/obese and metabolic syndrome participants	[22,23,24,25,26,27]

↑—increased; SFA—Saturated fatty acids; PUFA—Polyunsaturated fatty acids; MUFA—Monounsaturated fatty acids; SMZ—crosslinked alginate matrix; AMZ—alginate matrix.
